# Virological response and resistances over 12 months among HIV-infected children less than two years receiving first-line lopinavir/ritonavir-based antiretroviral therapy in Cote d’Ivoire and Burkina Faso: the MONOD ANRS 12206 cohort

**DOI:** 10.7448/IAS.20.01.21362

**Published:** 2017-04-25

**Authors:** Clarisse Amani-Bosse, Désiré Lucien Dahourou, Karen Malateste, Madeleine Amorissani-Folquet, Malik Coulibaly, Sophie Dattez, Arlette Emieme, Mamadou Barry, Christine Rouzioux, Sylvie N’gbeche, Caroline Yonaba, Marguerite Timité-Konan, Véronique Mea, Sylvie Ouédraogo, Stéphane Blanche, Nicolas Meda, Carole Seguin-Devaux, Valériane Leroy, for the MONOD ANRS 12206 Study Group

**Affiliations:** ^a^ PACCI Programme, Site ANRS, MONOD Project, Abidjan, Côte d’Ivoire; ^b^ MONOD Project, ANRS 12206, Centre de Recherche Internationale pour la Santé, Ouagadougou, Burkina Faso; ^c^ Clinical Research department, Centre Muraz, Bobo-Dioulasso, Burkina Faso; ^d^ Inserm, Unité U1219, Université Bordeaux, Bordeaux, France; ^e^ Pediatric Department, Centre Hospitalier Universitaire of Cocody, Abidjan, Côte d’Ivoire; ^f^ Virology departement, Laboratory CeDReS, Abidjan, Côte d’Ivoire; ^g^ Laboratory, Centre Hospitalier Universitaire de Ouagadougou, Ouagadougou, Burkina Faso; ^h^ EA 7327, Laboratoire de Virologie, Université Paris Descartes, CHU Necker, France; ^i^ CePReF-enfants, Abidjan, Côte d’Ivoire; ^j^ Pediatric Department, Centre Hospitalier Universitaire Yalgado Ouédraogo, Ouagadougou, Burkina Faso; ^k^ Pediatric Department, Centre Hospitalier Universitaire de Yopougon, Abidjan, Côte d’Ivoire; ^l^ Pediatric Department, Centre Hospitalier Universitaire Charles de Gaulle, Ouagadougou, Burkina Faso; ^m^ EA 8, Université-Paris Descartes, Paris, France; ^n^ Immunology, Hematology, Rhumatologie Unit, Hopital Necker Enfants Malades-Assistance Publique Hopitaux de Paris, Paris, France; ^o^ Public Health department, University of Ouagadougou, Ouagadougou, Burkina Faso; ^p^ Department of Infection and Immunity, Luxembourg Institute of Health, Luxembourg; ^q^ Inserm, UMR1027, Université Paul Sabatier Toulouse 3, Toulouse, France

**Keywords:** HIV, children, early antiretroviral treatment, lopinavir, treatment outcomes, cohort, West Africa

## Abstract

**Introduction**: Lopinavir/ritonavir-based antiretroviral therapy (ART) is recommended for all HIV-infected children less than three years. However, little is known about its field implementation and effectiveness in West Africa. We assessed the 12-month response to lopinavir/ritonavir-based antiretroviral therapy in a cohort of West African children treated before the age of two years.

**Methods**: HIV-1-infected, ART-naive except for a prevention of mother-to-child transmission (PMTCT), tuberculosis-free, and less than two years of age children with parent’s consent were enrolled in a 12-month prospective therapeutic cohort with lopinavir/ritonavir ART and cotrimoxazole prophylaxis in Ouagadougou and Abidjan. Virological suppression (VS) at 12 months (viral load [VL] <500 copies/mL) and its correlates were assessed.

**Results**: Between May 2011 and January 2013, 156 children initiated ART at a median age of 13.9 months (interquartile range: 7.8–18.4); 63% were from Abidjan; 53% were girls; 37% were not exposed to any PMTCT intervention or maternal ART; mother was the main caregiver in 81%; 61% were classified World Health Organization Stage 3 to 4. After 12 months on ART, 11 children had died (7%), 5 were lost-to-follow-up/withdrew (3%), and VS was achieved in 109: 70% of children enrolled and 78% of those followed-up. When adjusting for country and gender, the access to tap water at home versus none (adjusted odds ratio (aOR): 2.75, 95% confidence interval (CI): 1.09–6.94), the mother as the main caregiver versus the father (aOR: 2.82, 95% CI: 1.03–7.71), and the increase of CD4 percentage greater than 10% between inclusion and 6 months versus <10% (aOR: 2.55, 95% CI: 1.05–6.18) were significantly associated with a higher rate of VS. At 12 months, 28 out of 29 children with VL ≥1000 copies/mL had a resistance genotype test: 21 (75%) had ≥1 antiretroviral (ARV) resistance (61% to lamivudine, 29% to efavirenz, and 4% to zidovudine and lopinavir/ritonavir), of which 11 (52%) existed before ART initiation.

**Conclusions**: Twelve-month VS rate on lopinavir/ritonavir-based ART was high, comparable to those in Africa or high-income countries. The father as the main child caregiver and lack of access to tap water are risk factors for viral failure and justify a special caution to improve adherence in these easy-to-identify situations before ART initiation. Public health challenges remain to optimize outcomes in children with earlier ART initiation in West Africa.

## Introduction

By the end of 2013, there were 3.2 million (2.9–3.5 million) children younger than 15 years living with HIV globally. Among these HIV-infected children, 2.9 million (2.6–3.2 million) were living in Sub-Saharan Africa [1]. Without access to antiretroviral therapy (ART), more than 50% will die by the age of two years [2].

In 2008, the children with HIV early antiretroviral therapy (CHER) trial demonstrated the efficacy of early ART in reducing mortality in HIV-infected infants treated before 12 weeks of age compared to those treated according to the 2006 World Health Organization (WHO) guidelines: 4% versus 16%, respectively [3]. Hence, the WHO recommended the initiation of ART in all HIV-infected children before the age of 12 months, regardless of their disease progression [4]; these recommendations were extended to the age of two years in 2010 [5] and then five years in 2013 [6]. Furthermore, systematic early infant diagnosis of HIV was recommended for all HIV-exposed children from the age of six weeks to initiate early ART in those diagnosed as HIV infected [7]. Despite these recommendations, early ART initiation in HIV-infected children remains challenging particularly in West Africa [8,9], and this is mainly due to a late infant diagnosis among HIV-exposed infants [10–12]. In 2013, in Sub-Sahara Africa, only 24% (22–26%) of eligible HIV-infected children were receiving ART [1].

Regardless of previous exposure to prevention of mother-to-child transmission (PMTCT) of HIV, studies have shown that treatment failure at 24 weeks is more likely in children starting nevirapine-based ART than in those starting lopinavir/ritonavir (LPV/r)-based ART [3,13]. Thus, the WHO 2013 guidelines recommended that all children below the age of three years start LPV/r-based ART when feasible [6]. However, the availability of LPV/r remains a challenge in low-income countries [14]. The available oral syrup formulation of LPV/r in infants has cold chain requirements. The syrup is unpalatable, with the potential for suboptimal adherence. Since 2015, oral pellets received approval for use in children and do not require a cold chain; however, their effectiveness still needs to be assessed in Africa [15]. However, little is known about the field implementation and effectiveness of starting a LPV/r-based regimen in eligible HIV-infected children, especially in West Africa. In 2010, we implemented a randomized clinical trial to assess a simplification strategy of a LPV/r-based ART started in children younger than two years for at least 12 months [9]. In this study, we assessed the 12-month clinical, immunological, and virological response to LPV/r-based ART over the pre-randomization prospective cohort of children treated before the age of two years in Ouagadougou, Burkina Faso, and Abidjan, Côte d’Ivoire, and investigated its correlates.

## Methods

### Study design

The MONOD-ANRS-12206 study is an international, non-inferiority, open-label, phase 3, randomized clinical trial conducted in Ouagadougou, Burkina-Faso, and Abidjan, Côte d’Ivoire (ClinicalTrial.gov registry number: NCT01127204). Study sites were the Abobo-Avocatier urban health clinic, the CePReF-enfant and the Yopougon and Cocody University Hospitals in Abidjan, and the Yalgado Ouédraogo and the Charles de Gaulle University Hospitals in Ouagadougou [9,16]. The present study refers to the initial 12-month ART therapeutical cohort conducted in the pre-randomization phase. The protocol was approved by the “Comité d’Ethique pour la Recherche en Santé du Burkina Faso”, and the “Comité National d’Ethique et de la Recherche en Côte d’Ivoire”.

### Participants

All HIV-1-infected children (confirmed by a positive HIV-1DNA polymerase chain reaction (PCR) on blood sample), less than two years old, ART naive except for PMTCT exposure, and whose parents agreed to participate in the MONOD ANRS-12206 project were enrolled in a therapeutic cohort between May 2011 and February 2013 in the participating healthcare facilities. All children enrolled started ART with abacavir (ABC, 20 mg/mL, 8 mg/kg every 12 h) or zidovudine (ZDV, 10 mg/mL, 4 mg/kg every 12 h), lamivudine (3TC, syrup 10 mg/mL, 4 mg/kg every 12 h), and LPV/r (syrup 80/20 mg/mL, 12 mg/kg every 12 h) with cotrimoxazole (sulfamethoxazole, 20 mg/kg + trimethoprim, 4 mg/kg once-daily) as prophylaxis against opportunistic infections. Comprehensive counselling regarding treatment adherence was provided to caregivers. Children with either known intolerance to one of the drugs, who co-infected with HIV-2, with haemoglobin less than 7 g/dL, with neutrophils less than 750/mm^3^, with creatinine above five times the normal threshold, or with aspartate aminotransferase or alanine aminotransferase above five times the normal threshold were excluded. Children with tuberculosis infection at inclusion received once-daily efavirenz (EFV)-based ART and were excluded from this study. The drugs were provided by the national AIDS programmes under the responsibility of the country coordinating centres in charge of supplies and qualification of the batches.

### Sample size

Based on our sample size estimation to conduct the MONOD trial [16], we expected to recruit 146 children in virological success. As virological data were not available from the field yet at the time of our protocol, we expected a 90% suppression rate on LPV/r, according to the Yeni report 2008 [17]. Thus, assuming a 90% virological suppression rate among survivors at 12 months on LPV/r, we planned to recruit 162 children in the initial LPV/r-based cohort.

### Procedures

Patient’s data were collected prospectively using standardized questionnaires at their first contact, their pre-inclusion visit (four weeks prior to ART initiation), inclusion visit (at ART initiation), and monthly visits until 12 months. The pre-inclusion visit included informed consent, an interview to assess medical history (PMTCT exposure), a complete clinical examination (weight, height, and WHO clinical staging), tuberculosis screening (chest x-ray), a standard blood test (haematology, creatinine, urea, aspartate contre-transfert (AST), alanine-amino-transferase (ALT), total bilirubin, alkaline phosphatase (ALP), glucose, alkaline phosphatases, amylase, lipase, lipid assessment (total cholesterol, triglycerides, low-density lipoprotein (LDL) cholesterol, high-density lipoprotein (HDL) cholesterol, and very-low-density lipoprotein (VLDL)), a CD4 lymphocyte subpopulation count (percentage and absolute count), and a qualitative DNA PCR to confirm the child’s HIV status. In case of symptoms suggesting tuberculosis (prolonged fever, chronic cough, recent malnutrition, or failure of classic antibiotics for an infectious syndrome), the diagnosis was completed with a tuberculin skin test, gastric lavage on three consecutive days, and stool examination for *Mycobacterium tuberculosis*. CD4 count was performed using FACScan flow cytometer. HIV RNA viral loads were measured with real-time PCR using a commercial assay (Generic HIV Charge Virale, Biocentric, France). Blood samples were repeated at 3, 6, 9, and 12 months post-ART initiation and tested for HIV RNA, and a complete blood count was performed. CD4 and standard blood tests were repeated at 6 and 12 months post-ART initiation. Children were examined by a paediatrician monthly and whenever medically warranted. At each visit, when the drugs were given to families, therapeutic education was conducted systematically first by the assistant pharmacist and then by the social worker.

HIV-1 genotypic resistance testing was performed before ART initiation and at 12-month post-ART in case of virological failure when HIV-1 RNA ≥1000 copies/mL, either in Abidjan for samples collected in Côte d’Ivoire or in Luxembourg for samples collected in Burkina Faso. The ANRS consensus techniques (http://www.hivfrenchresistance.org) for genotyping protease and reverse-transcriptase (RT) genes were used. Sequences were edited with Bio-Edit sequence Alignment Editor (version 7.0) and trees constructed with Mega 4. Relevant drug-resistance mutations were interpreted according to the Stanford University HIV Drug Resistance Database (HIVdb Program, http://hivdb.stanford.edu) and the ANRS-v24 interpretation rule (available at: http://www.hivfrenchresistance.org/2011/Algo-2011.pdf). HIV-1 subtypes were assigned using REGA (http://www.bioafrica.net/rega-genotype/html/index.html) and COMET (http://comet.retrovirology.lu) HIV-1 subtyping tools against HIV-1 group M sequences from Genbank (http//www.ncbi.nlm.nih.gov/Genbank/index.html) as a reference.

### Statistical analysis

The main outcome measured was viral suppression (VS) defined as one single HIV-1 RNA <500 copies/mL after 12 months on ART. The 500 copies/mL threshold was chosen as this measurement was the validated threshold in both country laboratories in 2011. This single viral load measurement per patient was aimed to be operational and in line with the 2016 WHO recommendations [18]. We compared baseline demographic, clinical, and biological characteristics between countries where study took place using Student *t*-test for continuous or a Chi-square or Fisher’s exact test for categorical variables. Using the Kaplan–Meier method, we estimated the cumulative probability of reaching the first viral load <500 copies/mL by 12 months on ART. Follow-up time was censored at 12 months of follow-up or at time of last visit for children lost-to-follow-up or date of death for children who died. Independent variables were characteristics at pre-enrolment and at ART initiation: study country, age groups, gender, first-line nucleoside reverse-transcriptase inhibitor (NRTI) backbone, caregiver, access to electricity, access to tap water and fridge, history of ART drug exposure, anthropometric *z*-scores (analysed with WHO Anthro Software, version 3.2.2, January 2011), WHO clinical stage, viral load, and the difference in CD4 percentage between ART initiation and 6 month post-ART (referred to as CD4 delta).

We analysed the correlates of VS at twelth month, using a multivariate logistic regression including the variables above which were associated in the univariate analysis with *p* < 0.25. Then, we conducted a stepwise descendant analysis. Variables were retained in the final model if significantly associated with VS (*p* < 0.05) or their exclusion led to changes in the estimated odds ratio of other covariates by more than 10%. Final results were adjusted for country and gender to account for differences between study countries. All analyses were performed using SAS 9.1.3 (Cary, NC, USA).

## Results

### Cohort profile and baseline characteristics

**Figure 1.**
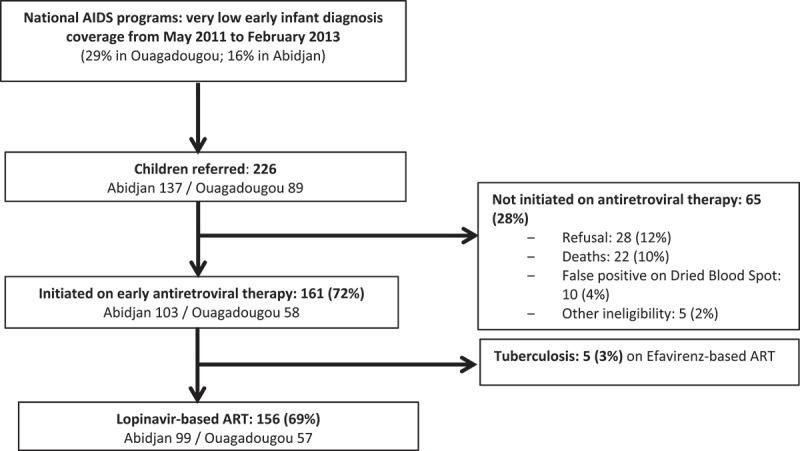
MONOD ANRS 12206 initial cohort profile, Abidjan, Côte d’Ivoire, Ouagadougou, Burkina Faso, 2011–2015.

Between May 2011 and January 2013, 226 children less than two years, first diagnosed as HIV-1 infected by HIV DNA PCR performed on dried blood spot, were referred to MONOD project clinics. Among these children, 65 (28%) did not meet inclusion criteria; 5 (2%) were co-infected with tuberculosis and initiated EFV -based therapy and thus excluded; and 156 (69%) initiated a LPV/r-based therapy and were included (Figure 1). The full inclusion process is described elsewhere [9].

Characteristics of the 156 children who initiated LPV/r-based therapy are shown in Table 1. Overall, 53% were girls; the median age at ART initiation was 13.9 months (interquartile range (IQR): 7.8–18.4). Children in Ouagadougou were significantly older by two months than those in Abidjan. The primary caregiver was the biological mother for 81% of the children; the biological father was the primary caregiver for 10% and the legal guardian for the remaining 9%. The proportion cared for by the biological father or legal guardian was significantly higher in Ouagadougou compared to Abidjan. In Abidjan, children’s families were significantly more likely to have tap water, electricity, and a fridge at home compared to those in Ouagadougou. Overall, 37% of the children were not exposed to any kind of PMTCT intervention or maternal ART, and this proportion was significantly higher in Ouagadougou compared to in Abidjan. At ART initiation, children from Ouagadougou had significantly more advanced HIV disease compared to those from Abidjan: they were more likely to be classified WHO clinical Stage 3 or 4, had lower anthropometric parameter *z*-scores, had lower CD4 percentage, and had higher viral load. The first-line NRTI backbone was ZDV-3TC for 91% of the children.

**Table 1.** Baseline characteristics according to site of the 156 HIV-infected children included in the ANRS 12206 MONOD cohort (Abidjan, Ouagadougou, May 2011 to February 2013).

Characteristics	Total,*N* =156	Abidjan,*N* =99	Ouagadougou,*N* =57	*p*-Value
**Pre-trial characteristics**				
Median age (months) at ART initiation (IQR)	13.9 (7.8–18.4)	13.3 (6.7–18.3)	15.4 (10.4–20.9)	0.02
<12 months, *n* (%)	65 (41.7)	46 (46.5)	19 (33.3)	0.11
≥12 months, *n* (%)	91 (58.3)	53 (53.5)	38 (66.7)	
Girl, *n* (%)	82 (52.6)	49 (49.5)	33 (57.9)	0.31
Father or other as main caregiver, *n* (%)	29 (18.6)	10 (10.1)	19 (33.3)	<0.01
Father informed of the child HIV status	100 (64.1)	63 (63.6)	37 (64.9)	0.87
Both parents alive, *n* (%)	136 (87.2)	81 (81.8)	55 (96.5)	<0.01
Individual housing, *n* (%)	82 (52.6)	46 (46.5)	36 (63.2)	0.04
Tap water at home, *n* (%)	105 (67.3)	84 (84.8)	21 (36.8)	<0.01
Electricity at home, *n* (%)	116 (74.4)	94 (94.9)	22 (38.6)	<0.01
Fridge at home, *n* (%)	35 (22.4)	28 (28.3)	7 (12.3)	0.02
Ever breastfed since birth, *n* (%)	136 (87.2)	84 (84.8)	52 (91.2)	0.25
Breastfeeding duration for those breastfed (months), median (IQR)	14.4 (7.7–20.9)	12.0 (6.2–16.1)	21.5 (14.3–25.2)	<0.01
**History of antiretroviral drug exposure**, *n* (%)				0.02
Prenatal maternal ART	19 (12.2)	17 (17.2)	2 (3.5)	
PMTCT and postnatal maternal ART	11 (7.1)	5 (5.1)	6 (10.5)	
PMTCT only	50 (32.1)	29 (29.3)	21 (36.8)	
Postnatal maternal ART only	18 (11.5)	15 (15.2)	3 (5.3)	
No previous exposure to any PMTCT or maternal ART	58 (37.2)	33 (33.3)	25 (43.9)	
**At child’s ART initiation**				
*z*-score, mean (SD)				
Weight-for-age	−2.4 (1.7)	−1.9 (1.6)	−3.1 (1.6)	<0.01
Height-for-age	−2.2 (1.9)	−1.9 (1.9)	−2.9 (1.6)	<0.01
Weight-for-height	−1.6 (1.5)	−1.2 (1.4)	−2.2 (1.5)	<0.01
WHO stage, *n* (%)				<0.01
Stage 1 or 2	61 (39.1)	54 (54.5)	7 (12.3)	
Stage 3 or 4	95 (60.9)	45 (45.5)	50 (87.7)	
Median haemoglobin (g/dL) (IQR)	9.1 (8.4–9.9)	9.4 (8.6–10.2)	8.5 (7.9–9.6)	<0.01
Median CD4 % (IQR)	20.0 (13.7–26.7)	21.3 (15.6–29.2)	17.9 (11.4–25.7)	0.03
Immunodeficiency for age, *n* (%)				0.51
None	18 (11.5)	14 (14.1)	4 (7.0)	
Mild	32 (20.5)	21 (21.2)	11 (19.3)	
Severe	104 (66.7)	63 (63.6)	41 (71.9)	
Missing	2 (1.3)	1 (1.0)	1 (1.8)	
Mean viral load (log_10_ copies/mL) (SD)	6.2 (1.0)	6.0 (1.0)	6.5 (0.9)	<0.01
Viral load ≥6 log_10_ copies/mL, *n* (%)	93 (59.6)	53 (53.5)	40 (70.2)	0.04
First-line NRTI backbone, *n* (%)				0.52
ZDV-3TC	142 (91.0)	89 (89.9)	53 (93.0)	
ABC-3TC	14 (9.0)	10 (10.1)	4 (7.0)	
Ever start cotrimoxazole, *n* (%)	154 (98.7)	97 (98.0)	57 (100.0)	0.53

ART: highly active antiretroviral therapy; PMTCT: prevention of mother-to-child transmission; SD: standard deviation; IQR: interquartile range; severe immunodeficiency for age: CD4 <25% if aged less than two years, CD4 <20% if aged two years or older; mild immunodeficiency for age: CD4 between 25% and 35% if aged less than two years, CD4 between 20% and 35% if aged two years or older; no immunodeficiency for age if CD4 >35%; ZDV: zidovudine; 3TC: lamivudine; ABC: abacavir; WHO: World Health Organization; NRTI: nucleoside reverse-transcriptase inhibitor.

### Clinical, immunological, and virological response to ART

Over the first 12 months on ART, 140 (90%) were followed-up 12 months, 11 infants died (7%), and 5 were lost-to-follow-up or withdrew (3%) (Table 2). Among the 11 deaths, 9 occurred within the first three months on ART, before viral load was measured, and the remaining 2 occurred 10 months after ART initiation: one had undetectable VL for six months prior to death. The median time to death was 18 days after inclusion (IQR: 3–333). The causes of death were gastroenteritis (*n* =5; 46%), severe malnutrition (*n* =3; 27%), respiratory infection (*n* =1; 9%), anaemia (*n* =1; 9%), and metabolic acidosis (*n* =1; 9%).

**Table 2.** Twelve-month outcome of the 156 HIV-infected children with LPV-based ART in the ANRS 12206 MONOD cohort according to site (Abidjan, Ouagadougou, May 2011to February 2013).

Outcomes	Total, *N* = 156	Abidjan, *N* = 99	Ouagadougou, *N* = 57	*p*-Value
**Immunological outcomes**				
**6 months**				
Median CD4% (IQR)	30.3 (24.1–37.0)	31.7 (26.5–37.0)	27.4 (21.8–36.5)	0.07
Delta CD4% M0–M6, *n* (%)				0.06
<10%	76 (48.7)	52 (52.5)	24 (42.1)	
≥10%	64 (41.0)	41 (41.4)	23 (40.4)	
Missing	16 (10.3)	6 (6.1)	10 (17.5)	
**12 months**				
Median CD4% (IQR)	31.4 (26.1–39.0)	31.4 (26.3–38.3)	31.4 (25.3–41.3)	0.63
Immunodeficiency for age^a^, *n* (%)				0.43
None	57 (36.5)	37 (37.4)	20 (35.1)	
Mild	66 (42.3)	45 (45.5)	21 (36.8)	
Severe	14 (9.0)	7 (7.1)	7 (12.3)	
Missing	19 (12.2)	10 (10.1)	9 (15.8)	
*z*-score, mean (SD)				
Weight-for-age	−1.5 (1.2)	−1.3 (1.0)	−1.8 (1.5)	0.03
Height-for-age	−1.8 (1.3)	−1.6 (1.2)	−2.1 (1.5)	0.02
Weight-for-height	−0.7 (1.1)	−0.6 (0.9)	−1.0 (1.5)	0.14
**Virological outcomes at 12 months**, *n* (%)				0.16^b^
Virological success (VL <500 copies/mL)	109 (69.9)	73 (73.7)	36 (63.2)	
Virological failure 500 ≥VL<1000 copies/mL	2 (1.3)	1 (1.0)	1 (1.8)	
Virological failure VL≥1000 copies/mL	29 (18.6)	17 (17.2)	12 (21.1)	
Death	11 (7.1)	5 (5.1)	6 (10.5)	
Loss to follow-up or withdrawal	5 (3.2)	3 (3.0)	2 (3.5)	

^a^Severe immunodeficiency for age: CD4 <25% if aged less than two years, CD4 <20% if aged two years or older; mild immunodeficiency for age: CD4 between 25% and 35% if aged less than two years, CD4 between 20% and 35% if aged two years or older; No immunodeficiency for age if CD4 >35%. ^b^Virological success versus virological failure or death or loss to follow-up. SD: standard deviation.

Among the 156 children included, the Kaplan–Meier cumulative probability of reaching a first VL measurement <500 copies/mL was 0.47 at 3 months, 0.81 at 6 months, 0.88 at 9 months, and 0.92 at 12 months. After 12 months on ART, the proportion of VS was 0.70 (95% confidence interval (CI): 0.62–0.77) of all children enrolled and 0.78 (95% CI: 0.71–0.85) of those followed-up. There was no statistically significant difference in VS between the two countries (Table 2).

The median CD4 percentage increased to 30.3 (IQR: 24.1–37.0) after six months on ART and to 31.4 (IQR: 26.1–39.0) at 12 months. Between baseline and 12 months post-ART, mean weight-for-age *z*-score (WAZ), height-for-age *z*-score, and weight-for-height *z*-score have increased from −2.4 (standard deviation (SD): 1.7) to −1.5 (SD: 1.2), −2.2 (SD: 1.9) to −1.8 (SD: 1.3), and −1.6 (SD: 1.5) to −0.7 (SD: 1.1), respectively.

During the first 12 months on ART, 37 (22.4%) children had at least one hospitalization or clinical severe adverse event. These were more common in children in Ouagadougou compared to those in Abidjan (*p* = 0.04). Ten (6.4%) children changed antiretroviral drug (substituting zidovudine with abacavir) because of severe adverse effects linked to anaemia. There were no significant differences in daytime or night-time sleeping disorders, declared by caregivers, between the two study countries (*p* = 0.98). Neutropenia was the most commonly reported grade 3 or 4 biological adverse event and was significantly more frequent in Abidjan than in Ouagadougou (*p* = 0.02). Hyperbilirubinaemia was more frequent in Ouagadougou than in Abidjan (*p* = <0.01). There were no significant differences between both countries for other grade 3 or 4 biological adverse events (Table 3).

**Table 3.** Incidence of grade 3 and 4 adverse events during 12 months of cohort of the 156 HIV-infected children included in the ANRS 12206 MONOD study according to study countries (Abidjan, Ouagadougou, February 2013 t oApril 2015).

	Total,	Abidjan,	Ouagadougou,	
Outcomes	*N* =156	*N* =99	*N* =57	*p*-Value
**Serious adverse events (SAEs)**				
Hospitalizations and clinical SAE	35 (22.4)	17 (17.2)	18 (31.6)	0.04
Toxicity causing ART modification	10 (6.4)	8 (8.1)	2 (3.5)	0.33
Sleeping disorders	44 (28.2)	28 (28.3)	16 (28.1)	0.98
**Specific biological adverse events**				
Anaemia, grade 3 and 4	8 (5.1)	5 (5.0)	3 (5.3)	1.00
Neutropenia, grade 3 and 4	28 (17.9)	23 (23.2)	5 (8.8)	0.02
Thrombopenia, grade 3 and 4	3 (1.9)	3 (3.0)	0 (0.0)	0.30
Hyperglycaemia, grade 3 and 4	0 (0.0)	0 (0.0)	0 (0.0)	–
Hypoglycaemia, grade 3 and 4	2 (1.3)	0 (0.0)	2 (3.5)	0.13
Hypercholesterolaemia, grade 3	3 (1.9)	3 (3.0)	0 (0.0)	0.30
Hypertriglyceridaemia, grade 3 and 4	0 (0.0)	0 (0.0)	0 (0.0)	–
Hypercreatininaemia, grade 3 and 4	2 (1.3)	1 (1.0)	1 (1.7)	1.00
Hypertransaminasemia AST or ALT, grade 3 and 4	1 (0.6)	0 (0.0)	1 (1.7)	0.36
Hyperbilirubinaemia, grade 3 and 4	8 (5.1)	1 (1.0)	7 (12.3)	<0.01
Hyperamylasaemia, grade 3 and 4	9 (5.8)	7 (7.1)	2 (3.5)	0.49
Hyperlipasaemia, grade 3 and 4	0 (0.0)	0 (0.0)	0 (0.0)	–

AST: aspartate contre-transfert; ALT: alanine-amino-transferase; ART: antiretroviral therapy.

### Correlates of viral suppression

In univariate analyses, study country, sex, first-line NRTI backbone, access to tap water, having electricity and a fridge at home, the main caregiver, having disclosed the child’s HIV status to the father, baseline, WHO clinical stage, WAZ, and the CD4 percentage delta between inclusion and six months on ART were all associated with VS at the 12-month visit with a *p*-value of <0.25 (Table 4).

**Table 4.** Description of factors associated with the 12-month virological success (<500 copies/mL) in children infected with HIV and treated early before the age of two years in the MONOD ANRS 12206 cohort, univariate analysis (Abidjan, Ouagadougou, May 2011 to February 2013).

	Total,	Virological success (<500 copies/mL),	Virological failure,	
	*N* = 156	*N* =109	*N* = 47	*p*-Value
Country				0.16
Abidjan	99 (63.5)	73 (67.0)	26 (55.3)	
Ouagadougou	57 (36.5)	36 (33.0)	21 (44.7)	
Age at ART initiation				0.57
<12 months	65 (41.7)	47 (43.1)	18 (38.3)	
≥12 months	91 (58.3)	62 (56.9)	29 (61.7)	
Gender				0.19
Girl	82 (52.6)	61 (56.0)	21 (44.7)	
Boy	74 (47.4)	48 (44.0)	26 (55.3)	
ART				0.23
ZDV + 3TC + LPV/r	142 (91.0)	97 (89.0)	45 (95.7)	
ABC + 3TC + LPV/r	14 (9.0)	12 (11.0)	2 (4.3)	
Housing of children				0.65
Individual housing	82 (52.6)	56 (51.4)	26 (55.3)	
Common court	74 (47.4)	53 (48.6)	21 (44.7)	
Tap water at home				0.01
No	51 (32.7)	29 (26.6)	22 (46.8)	
Yes	105 (67.3)	80 (73.4)	25 (53.2)	
Electricity at home				0.24
No	40 (25.6)	25 (22.9)	15 (31.9)	
Yes	116 (74.4)	84 (77.1)	32 (68.1)	
Fridge at home				0.06
No	121 (77.6)	80 (73.4)	41 (87.2)	
Yes	35 (22.4)	29 (26.6)	6 (12.8)	
Main caregiver for children				0.05
Mother main caregiver	127 (81.4)	93 (85.3)	34 (72.3)	
Father/other in charge of care	29 (18.6)	16 (14.7)	13 (27.7)	
Father informed of HIV status of the child				0.08
No	56 (35.9)	44 (40.4)	12 (25.5)	
Yes	100 (64.1)	65 (59.6)	35 (74.5)	
Vital status of parents				0.29
Both parents alive	136 (87.2)	93 (85.3)	43 (91.5)	
At least one of the two deceased	20 (12.8)	16 (14.7)	4 (8.5)	
History of antiretroviral drug exposure				0.36
No previous exposure to any PMTCT or ART	58 (37.2)	39 (35.8)	19 (40.4)	
Prenatal maternal ART	19 (12.2)	11 (10.1)	8 (17.0)	
Exposure to PMTCT only	50 (32.1)	35 (32.1)	15 (31.9)	
Exposure to postnatal maternal ART only	18 (11.5)	14 (12.8)	4 (8.5)	
PMTCT and postnatal maternal ART	11 (7.1)	10 (9.2)	1 (2.1)	
WHO clinical stage at ART initiation				0.08
Stages 1, 2, 3	123 (78.8)	90 (82.6)	33 (70.2)	
Stage 4	33 (21.2)	19 (17.4)	14 (29.8)	
*z*-score WAZ at ART initiation				0.09
Severe	55 (35.3)	33 (30.3)	22 (46.8)	
Moderate	28 (17.9)	23 (21.1)	5 (10.6)	
Normal	73 (46.8)	53 (48.6)	20 (42.6)	
*z*-score HAZ at ART initiation				0.25
Severe	46 (29.5)	28 (25.7)	18 (38.3)	
Moderate	34 (21.8)	24 (22.0)	10 (21.3)	
Normal	76 (48.7)	57 (52.3)	19 (40.4)	
Viral load (log_10_ copies/mL) at ART initiation				0.32
≥6 log_10_/missing	97 (62.2)	65 (59.6)	32 (68.1)	
<6 log_10_	59 (37.8)	44 (40.4)	15 (31.9)	
Delta %CD4 M0–M6				<0.01
<10%	76 (48.7)	52 (47.7)	24 (51.1)	
≥10%	64 (41.0)	54 (49.5)	10 (21.3)	
Missing	16 (10.3)	3 (2.8)	13 (27.7)	

WAZ: weight-for-age *z*-score; HAZ: height-for-age *z*-score; ZDV: zidovudine; 3TC: lamivudine; ABC: abacavir; LPV/r: lopinavir/ritonavir; ART: antiretroviral therapy; PMTCT: prevention of mother-to-child transmission.

In the final analysis, adjusted for study country and sex, access to tap water at home (adjusted odds ratio (aOR): 2.75, 95% CI: 1.09–6.94), having the biological mother as the main caregiver (aOR: 2.82, 95% CI: 1.03–7.71), and a ≥10% increase of CD4 percentage within the first six months on ART (aOR: 2.55, 95% CI: 1.05–6.18) were all significantly associated with a higher rate of VS at 12 months on ART (Table 5).

**Table 5.** Factors associated with virological success at 12 months in children infected with HIV and treated early before the age of two years in the MONOD ANRS 12206 cohort, regression analysis (Abidjan, Ouagadougou, May 2011 to February 2013)

	Univariate analyses			Full model			Adjusted analyses		
	OR	CI (95%)	*p*	OR	CI (95%)	*p*	aOR	CI (95%)	*p*
Abidjan versus Ouagadougou	1.64	(0.81–3.30)	0.17	0.71	(0.21–2.34)	0.57	0.66	(0.25–1.77)	0.41
Age at ART initiation <12 months versus ≥12 months	1.22	(0.61–2.46)	0.57	–	–		–	–	
Girl versus boy	1.57	(0.79–3.13)	0.20	2.60	(1.07–6.29)	0.03	2.03	(0.90–4.60)	0.09
ART			0.19			0.05	–	–	
ZDV + 3TC + LPV/r	Ref.	–		Ref.	–				
ABC + 3TC + LPV/r	2.78	(0.60–12.96)		6.86	(1.03–45.77)				
Individual housing versus common court	0.85	(0.43–1.70)	0.65	–	–		–	–	
Tap water at home versus no	2.43	(1.19–4.95)	0.01	2.94	(1.03–8.38)	0.04	2.75	(1.09–6.94)	0.03
Electricity at home versus no	1.57	(0.74–3.36)	0.24	0.58	(0.15–2.17)	0.42	–	–	
Fridge at home versus no	2.48	(0.95–6.44)	0.06	2.65	(0.81–8.64)	0.11	–	–	
Mother as main caregiver versus father or other	2.22	(0.97–5.10)	0.06	2.82	(0.96–8.30)	0.06	2.82	(1.03–7.71)	0.04
Father informed of HIV status of the child versus no	0.51	(0.24–1.08)	0.08	0.53	(0.21–1.32)	0.17	–	–	
At least one of the two deceased parents versus both parents alive	1.85	(0.58–5.86)	0.30	–	–		–	–	
History of antiretroviral drug exposure			0.42	–	–		–	–	
No previous exposure to any PMTCT or ART	Ref.	–							
Prenatal maternal ART	0.67	(0.23–1.94)							
Exposure to PMTCT only	1.14	(0.50–2.57)							
Exposure to postnatal maternal ART only	1.70	(0.49–5.89)							
PMTCT and postnatal maternal ART	4.87	(0.58–40.89)							
WHO clinical stage at ART initiation			0.09	–	–		–	–	
Stage 1, 2, or 3	Ref.	–							
Stage 4	0.50	(0.22–1.10)							
*z*-score WAZ at ART initiation			0.10			0.68	–	–	
Severe	Ref.	–		Ref.	–				
Moderate	3.07	(1.01–9.28)		1.73	(0.45–6.67)				
Normal	1.77	(0.84–3.72)		1.38	(0.51–3.76)				
*z*-score HAZ at ART initiation			0.26	–	–		–	–	
Severe	Ref.	–							
Moderate	1.54	(0.60–3.97)							
Normal	1.93	(0.88–4.24)							
Viral load (log_10_ copies/mL) at ART initiation <6 log_10_ copies/mL versus ≥6 log_10_ copies/mL or missing	1.44	(0.70–2.97)	0.32	–	–		–	–	
Delta CD4% M0–M6			<0.01			<0.01			<0.01
<10%	Ref.	–		Ref.	–		Ref.	–	
≥10%	2.49	(1.09–5.72)		2.18	(0.86–5.52)		2.55	(1.05–6.18)	
Missing	0.11	(0.03–0.41)		0.08	(0.02–0.35)		0.08	(0.02–0.34)	

ART: antiretroviral therapy; OR: odds ratio; aOR: adjusted odds ratio; CI: confidence interval; Forced variables: country and gender; ZDV: zidovudine; 3TC: lamivudine; ABC: abacavir; LPV/r: lopinavir/ritonavir; PMTCT: prevention of mother-to-child transmission; WHO: World Health Organization; WAZ: weight-for-age *z*-score; HAZ: height-for-age *z*-score.

### Drug resistance

Among the 29 children with VL ≥1000 copies/mL at 12 months, 28 had genotypic resistance testing: 21 (75%) had a virus resistant to at least one drug and 11 (52%) existed prior to ART initiation and were classified as transmitted resistant virus (3 children were exposed to maternal PMTCT alone, 3 exposed to maternal ART initiation during breastfeeding, 2 exposed to both perinatal and postnatal ART, 2 unexposed, and one unknown). The most frequent resistance was to lamivudine (61%); 29% had resistance mutations to non-nucleoside reverse-transcriptase inhibitors (NNRTIs) and 4% had resistance mutations to both zidovudine and LPV/r (Table 6).

**Table 6.** Resistance profiles among children followed up at 12 months and in virological failure (*N* =28/31) ANRS 12206 MONOD cohort (Abidjan, Ouagadougou, May 2011 to February 2014)

	*N* = 28	Percentage
**No drug resistance mutation**	7	25
**Resistance to at least one drug by group**	21	75
**Resistance to one class**		
3TC	13	46
NNRTI: EFV/NVP/RPV/ETR	4	15
**Resistance to two classes**		
3TC/EFV/NVP	3	11
**Resistance to three classes (NRTI/NNRTI/PI)**		
AZT/3TC/EFV/NVP/SQV/LPVr/IDV	1	4

NRTI: nucleoside reverse-transcriptase inhibitors; AZT: zidovudine; 3TC: lamivudine; d4T: stavudine; NNRTI: non-nucleoside reverse-transcriptase inhibitors; EFV: efavirenz; NVP: nevirapine; ETR: etravirine; RPV: rilpivirine; PI: protease inhibitors; SQV: saquinavir; IDV: indinavir; LPV: lopinavir; R: ritonavir.

## Discussion

Our study reports for the first time in West African settings the 12-month response and its correlates to a LPV/r-based ART in a cohort of ART-naive HIV-1-infected children treated before two years of age. Despite a relatively late access to ART initiation, at an already advanced stage of HIV disease, high rates of VS can be achieved among young children who initiate protease-inhibitor-based ART. After 12 months on ART, 78% of those followed-up achieved VS. However, we also observed a high rate of HIV-related deaths reaching 7%, and occurring early after ART initiation, which we explain by the late access to ART. Access to tap water, having their biological mother as the primary caregiver, and an increase of CD4 percentage between ART initiation and six-month visit equal or greater than 10% were identified as correlates to the 12-month VS after ART initiation in this cohort. History of exposure to a PMTCT intervention was not associated with 12-month VS in this study.

First, we observed high early mortality after ART initiation that has already been described in other cohorts of HIV-infected children initiating ART [19–23]. In a South African cohort, mortality was higher, reaching 14% at 39 weeks, mainly related to tuberculosis, while in our study children with tuberculosis at inclusion were excluded [20]. This high rate of mortality is related to the delay in ART initiation [8], which is mainly due to the difficulty of early infant diagnosis, as already observed in other studies conducted in low-income countries [24,25]. Young HIV-infected children are particularly vulnerable to rapid HIV disease progression and early mortality [2]. In our cohort, median age was 13.9 months, with advanced HIV disease, low CD4 percentage, and a high mean viral load level of 6.2 log/mL at ART initiation. This emphasizes the need to reduce more significantly the delay to ART initiation among HIV-infected children to optimize their survival outcome.

Second, despite delayed access to ART, children experienced a high rate of VS reaching 78% among those surviving that was comparable to other settings, in Africa, as well as in high-income countries [26–29]. This rate of VS is also similar to the rate of 84% recorded in an LPV/r-treated cohort follow-up in South Africa [21] and of 73% recorded in an NNRTI-treated cohort in Uganda and Zimbabwe [23,27]. As reported in previous adult studies, rates of VS are expected to be higher in the “on-treatment analyses” than in the “intention-to-treat analyses” and also to increase as the viral RNA thresholds used to define suppression increases [30]. Again, earlier access to ART would probably have optimized this initial rate of VS. This highlights the fact that access to early infant diagnosis and early ART before the age of two years still remains challenging in real life in West African settings [31]. The scaling-up of recommended early ART remains a major public health challenge in resource-limited settings [10].

Third, at ART initiation, access to tap water at home was a determinant for VS, with a 2.75 higher rate at 12 months in our study. Lack of access to tap water at home reflects deprived socio-economic conditions, which could probably be the marker of a poor adherence leading to virological failure. We also showed the key role of the caregiver in the success of HIV-infected children treatment: the fact that the father or another person than the biological mother was the main child caregiver was associated with a significant lower VS rate compared to the mother. This result underlines that the mother has a leading role in child survival as already reported elsewhere [32]. Before ART initiation, access to tap water and not having the biological mother as the principal caregiver could therefore be two indicators of first-line ART failure, easy to verify, to strengthen their support to adherence and to monitor closely treatment response. We noticed in our study that the father is also more frequently in charge of the child in Ouagadougou than in Abidjan, and declared as the main caregiver, even if the mother is alive because in Burkina Faso the father is empowered to take the decisions about the children’s healthcare seeking and treatments. Consequently, they also accompanied more frequently their child to the medical visit, as already reported in the inclusion process [9].

Fourth, our results also show good immune recovery, with a 10% increase in CD4 percentage, from 20% at baseline to 30% in median at 6 months. This result is comparable to those of other cohorts of children receiving ART in Africa [23,33,34]. We did not find an association between VS and factors found in other studies such as age, gender, previous exposures to PMTCT or to maternal ART, baseline WHO stage, CD4 percentage, or baseline viral load [23,35–37]. But, we found that an increase of CD4 percentage between baseline and six months equal or greater than 10% doubled the odds of VS 12 months after ART initiation. This could be used as an early indicator to monitor the risk of virological failure in low-income countries where viral load is not widely available. We also found that LPV/r-based ART was well tolerated with few severe adverse events, most of which were clinical adverse events and hospitalizations mostly HIV related rather than drug related. We will further analyse adherence data collected using a four-day recall questionnaire of missed doses correlated to pharmacokinetic data to study the relationship with VS. Among children with virological failure, we recorded a high rate of resistance to at least one antiretroviral drug (75%), of whom 52% were acquired after ART initiation. This high rate is of particular concern in our setting where ART options are limited. This proportion is comparable with the pooled proportion of 90% of at least one detectable resistance mutation found in a systematic review of antiretroviral resistance data in children from developing countries [38]. As previously showed by other studies, we recorded a high rate of drug resistance to lamivudine and to NNRTIs [38–41]. As the preferred regimens for second line recommended by WHO for children older than 3 years is efavirenz-based ART [18, 42], these children with resistance to NNRTIs will be at higher risk of treatment failure when switching to  second-line regimens. We did not find any differences in VS between children with and without previous PMTCT exposure, and drug resistance to LPV/r was rare in our study. This reflects the high genetic barriers of LPV/r and its interest for first-line therapy in young children, consistent with others studies [29,43] and with WHO consolidated guidelines and recommendations [18]. Finally, the occurrence of triple-class resistance was exceptional in our study. Our study highlights the interest of a first-line LPV/r-based regimen in the context of multi-exposure to PMTCT and postnatal antiretroviral drugs in West African children that will be further investigated. In the context of early treatment for all HIV-infected children and increased risk of drug resistance with the duration of therapy [38,44,45], there is an urgent need to identify children not responding to ART as early as possible, to promote optimum adherence to ART in these children and their caregivers, to develop ART simplification strategies, and to develop new drugs and formulations to optimize the treatment of children with virological failure.

Our study however presents some limitations. First, we included clinics from urban settings only, with probably a better access to early infant diagnosis and ART compared to rural settings, where the delay in care could be even longer than that reported in our study. Second, the high rate of VS in our study was obtained in the context of clinical research involving all guarantees for optimal monitoring of the family, leading to an overestimation of the rate of VS compared to real-life routine ART programmes. Third, we were not able to report here the role of adherence in relation with the virological response. In the MONOD project, we used an adherence assessment survey based on the drug doses missed over the last four days before each visit; however, this method was found to be unreliable. Pharmacokinetic studies are ongoing and these data will be considered in, separate, subsequent analyses. Fourth, although the relatively small size of our cohort leads to a lack of statistical power in comparisons, we achieved very good follow-up quality with only 3% of children lost-to-follow-up or withdrawing. Despite these limitations, our findings are original and described for the first time a West African cohort of children treated with LPV/r-based therapy before the age of two years and documents the 12-month response.

In conclusion, in this West African cohort, we show that initiating a first-line LPV/r-based ART before the age of two is feasible and appropriate and a VS rate of 78% is achieved by 12 months. We identified two correlates to low VS, probably linked to poor adherence when the mother was not the main caregiver or if the family had no access to tap water. These markers could be easily notified at ART initiation, and reinforced monitoring could be offered to improve adherence and VS. We also report that over the 2011–2013 period, challenges still remain for improving early access to ART in HIV-infected children in West Africa. An earlier access combined with targeted interventions for those at risk for treatment failure will be helpful in optimizing the VS rate in West Africa and tend towards the 90% expected. Nevertheless, the development of a protease-inhibitor formulation easier to handle and more baby-friendly combined formulations remain also critical to achieve this 90% VS rate goal in this population [46].

## References

[1] UNAIDS Global report UNAIDS report on the global AIDS epidemic. Geneva: UNAIDS; 2013.

[2] Newell ML, Coovadia H, Cortina Borja M, Rollins N, Gaillard P, Dabis F, et al Mortality of infected and uninfected infants born to HIV-infected mothers in Africa: a pooled analysis. Lancet. 2004;364(9441):1236–13. 15464184 10.1016/S0140-6736(04)17140-7

[3] Violari A, Cotton MF, Gibb DM, Babiker AG, Steyn J, Madhi SA, et al Early antiretroviral therapy and mortality among HIV-infected infants. N Engl J Med. 2008;359(21):2233–44. 19020325 10.1056/NEJMoa0800971 PMC2950021

[4] World Health Organisation Report of the WHO Technical Reference Group, Paediatric HIV/ART Care Guideline Group Meeting. Revised treatment recommendations for infants Geneva: WHO; 2008 [cited 2008 410–11]. 41p]. Available from: http://www.who.int/hiv/pub/paediatric/WHO_Paediatric_ART_guideline_rev_mreport_2008.pdf

[5] World Health Organisation Antiretroviral therapy for HIV infection in infants and children: towards universal access. Recommendations for a public health approach. Revision 2010 Geneva: WHO; 2010 [cited 7 2010]. 206 p.]. Available from: http://whqlibdoc.who.int/publications/2010/9789241599801_eng.pdf 23741772

[6] World Health Organisation Consolidated guidelines on the use of antiretroviral drugs for treating and preventing HIV infection. Recommendations for a public health approach. Geneva: World Health Organization HIV/AIDS Department, 2013 [cited 2013 630] Report No. 24716260

[7] World Health Organisation Diagnosis of HIV infection in infants and children. Geneva: WHO, 2010 [cited 2010 713]. Report No.

[8] Leroy V, Malateste K, Rabie H, Lumbiganon P, Ayaya S, Dicko F, et al Outcomes of antiretroviral therapy in children in Asia and Africa: a comparative analysis of the IeDEA pediatric multiregional collaboration. J Acquir Immune Defic Syndr. 2013;62(2):208–19. 23187940 10.1097/QAI.0b013e31827b70bf PMC3556242

[9] Dahourou DL, Amorissani-Folquet M, Coulibaly M, Avit-Edi D, Meda N, Timite-Konan M, et al Missed opportunities of inclusion in a cohort of HIV-infected children to initiate antiretroviral treatment before the age of two in West Africa, 2011 to 2013. J Int AIDS Soc. 2016;19(1):20601. 27015798 10.7448/IAS.19.1.20601 PMC4808141

[10] Penazzato M, Revill P, Prendergast AJ, Collins IJ, Walker S, Elyanu PJ, et al Early infant diagnosis of HIV infection in low-income and middle-income countries: does one size fit all? Lancet Infect Dis. 2014;14(7):650–55. 24456814 10.1016/S1473-3099(13)70262-7

[11] Gupta A, Singh G, Kaushik P, Joshi B, Kalra K, Chakraborty S. Early diagnosis of HIV in children below 18 months using DNA PCR test – assessment of the effectiveness of PMTCT interventions and challenges in early initiation of ART in a resource-limited setting. J Trop Pediatr. 2013;59(2):120–26. 23221038 10.1093/tropej/fms063

[12] Ciaranello AL, Park J, Ramirez-Avila L, Freedberg KA, Walensky RP, Leroy V, Early infant HIV-1 diagnosis programs in resource-limited settings: opportunities for improved outcomes and more cost-effective interventions, BMC Med. 2011;9:59. 21599888 10.1186/1741-7015-9-59 PMC3129310

[13] Palumbo P, Lindsey JC, Hughes MD, Cotton MF, Bobat R, Meyers T, et al Antiretroviral treatment for children with peripartum nevirapine exposure. N Engl J Med. 2010;363(16):1510–20. 20942667 10.1056/NEJMoa1000931 PMC3021781

[14] Prendergast AJ, Penazzato M, Cotton M, Musoke P, Mulenga V, Abrams EJ, et al Treatment of young children with HIV infection: using evidence to inform policymakers. Plos Med. 2012;9(7):e1001273. 22910874 10.1371/journal.pmed.1001273 PMC3404108

[15] Kekitiinwa A, Musiime V, Thomason MJ, Mirembe G, Lallemant M, Nakalanzi S, et al Acceptability of lopinavir/r pellets (minitabs), tablets and syrups in HIV-infected children. Antiviral Therapy. 2016;21:579–85. 27128199 10.3851/IMP3054 PMC6029664

[16] Dahourou D, Amorissani-Folquet M, Malateste K, Amani-Bosse C, Coulibaly M, Devaux C, et al Efavirenz-based simplification after successful early LPV/r-based therapy in HIV-infected children in Burkina Faso and Côte d’Ivoire: the MONOD ANRS 12206 non inferiority randomised trial. BMC Med. Forthcoming 2017. 10.1186/s12916-017-0842-4 PMC5402051 28434406

[17] Yeni P Rapport du Groupe d’Experts 2008 sur la prise en charge médicale des patients infectées par le VIH, sous la direction du Pr Patrick Yeni 2008. Available from: http://www.sante-jeunesse-sports.gouv.fr/publications-documentation/publications-documentation-sante/rapports/rapport-du-groupe-experts-2008-prise-charge-medicale-patients-infectees-par-vih-sous-direction-du-pr-patrick-yeni.html

[18] World Health Organisation Consolidated guidelines on the use of antiretroviral drugs for treating and preventing HIV infection. Ecommendations for a public health approach – second edition Geneva: WHO, UNAIDS; 2016 [cited 2016 6]. Available from: http://www.who.int/hiv/pub/arv/arv-2016/en/27466667

[19] Bolton-Moore C, Mubiana-Mbewe M, Cantrell RA, Chintu N, Stringer EM, Chi BH, et al Clinical outcomes and CD4 cell response in children receiving antiretroviral therapy at primary health care facilities in Zambia. JAMA. 2007;298(16):1888–99. 17954540 10.1001/jama.298.16.1888

[20] Reddi A, Leeper SC, Grobler AC, Geddes R, France KH, Dorse GL, et al Preliminary outcomes of a paediatric highly active antiretroviral therapy cohort from KwaZulu-Natal, South Africa. BMC Pediatr. 2007;7:13. 17367540 10.1186/1471-2431-7-13 PMC1847430

[21] Reitz C, Coovadia A, Ko S, Meyers T, Strehlau R, Sherman G, et al Initial response to protease-inhibitor-based antiretroviral therapy among children less than 2 years of age in South Africa: effect of cotreatment for tuberculosis. J Infect Dis. 2010;201(8):1121–31. 20214476 10.1086/651454 PMC2946637

[22] Anaky MF, Duvignac J, Wemin L, Kouakoussui A, Karcher S, Toure S, et al Scaling up antiretroviral therapy for HIV-infected children in Cote d’Ivoire: determinants of survival and loss to programme. Bull World Health Organ. 2010;88(7):490–99. 20616968 10.2471/BLT.09.068015 PMC2897983

[23] Tukei VJ, Murungi M, Asiimwe AR, Migisha D, Maganda A, Bakeera-Kitaka S, et al Virologic, immunologic and clinical response of infants to antiretroviral therapy in Kampala, Uganda. BMC Pediatr. 2013; 13:42. 23536976 10.1186/1471-2431-13-42 PMC3616823

[24] Gupta A, Singh G, Kaushik P, Joshi B, Kalra K, Chakraborty S Early diagnosis of HIV in children below 18 months using DNA PCR test – assessment of the effectiveness of PMTCT interventions and challenges in early initiation of ART in a resource-limited setting. J Trop Pediatr. 2013;59(2):120–6. 23221038 10.1093/tropej/fms063

[25] Ciaranello AL, Park JE, Ramirez-Avila L, Freedberg KA, Walensky RP, Leroy V Early infant HIV-1 diagnosis programs in resource limited settings: opportunities for improved outcomes and more cost-effective interventions. BMC Med. 2011;9(1):59. 21599888 10.1186/1741-7015-9-59 PMC3129310

[26] Ruel TD, Kakuru A, Ikilezi G, Mwangwa F, Dorsey G, Rosenthal PJ, et al Virologic and immunologic outcomes of HIV-infected Ugandan children randomized to lopinavir/ritonavir or nonnucleoside reverse transcriptase inhibitor therapy. J Acquir Immune Defic Syndr. 2014;65(5):535–41. 24326597 10.1097/QAI.0000000000000071 PMC3999287

[27] Musoke P, Szubert AJ, Musiime V, Nathoo K, Nahirya-Ntege P, Mutasa K, et al Single-dose nevirapine exposure does not affect response to antiretroviral therapy in HIV-infected African children aged below 3 years. AIDS. 2015;29(13):1623–32. 26193705 10.1097/QAD.0000000000000749 PMC4833198

[28] Judd A Early antiretroviral therapy in HIV-1-infected infants, 1996–2008: treatment response and duration of first-line regimens. AIDS. 2011;25(18):2279–87. 21971357 10.1097/QAD.0b013e32834d614c PMC3433031

[29] Frange P, Briand N, Avettand-Fenoel V, Veber F, Moshous D, Mahlaoui N, et al Lopinavir/ritonavir-based antiretroviral therapy in human immunodeficiency virus type 1-infected naive children: rare protease inhibitor resistance mutations but high lamivudine/emtricitabine resistance at the time of virologic failure. Pediatr Infect Dis J. 2011;30(8):684–88. 21427626 10.1097/INF.0b013e31821752d6

[30] McMahon JH, Elliott JH, Bertagnolio S, Kubiak R, Jordan MR Viral suppression after 12 months of antiretroviral therapy in low- and middle-income countries: a systematic review. Bull World Health Organ. 2013;91(5):377–85E. 23678201 10.2471/BLT.12.112946 PMC3646348

[31] Coulibaly M, Meda N, Yonaba C, Ouedraogo S, Congo M, Barry M, et al Missed opportunities for early access to care of HIV-infected infants in Burkina Faso. Plos ONE. 2014;9(10):e111240. 25360551 10.1371/journal.pone.0111240 PMC4215985

[32] Newell M-L, Coovadia H, Cortina-Borja M, Rollins N, Gaillard P, Dabis F Mortality of infected and uninfected infants born to HIV-infected mothers in Africa: a pooled analysis. Lancet. 2004;364(9441):1236–43. 15464184 10.1016/S0140-6736(04)17140-7

[33] Janssen N, Ndirangu J, Newell ML, Bland RM Successful paediatric HIV treatment in rural primary care in Africa. Arch Dis Child. 2010;95(6):414–21. 19880392 10.1136/adc.2009.169367 PMC3181433

[34] Soeters HM, Sawry S, Moultrie H, Rie AV The effect of tuberculosis treatment on virologic and immunologic response to combination antiretroviral therapy among South African children. J Acquir Immune Defic Syndr. 2014;67(2):136–44. 25072611 10.1097/QAI.0000000000000284 PMC4162748

[35] Shiau S, Kuhn L, Strehlau R, Martens L, McIlleron H, Meredith S, et al Sex differences in responses to antiretroviral treatment in South African HIV-infected children on ritonavir-boosted lopinavir- and nevirapine-based treatment. BMC Pediatr. 2014;14:39. 24521425 10.1186/1471-2431-14-39 PMC3927631

[36] Estripeaut D, Mosser J, Doherty M, Acosta W, Shah H, Castano E, et al Mortality and long-term virologic outcomes in children and infants treated with lopinavir/ritonavir. Pediatr Infect Dis J. 2013;32(12):e466–72. 23799516 10.1097/INF.0b013e3182a09276 PMC3883902

[37] Lindsey JC, Hughes MD, Violari A, Eshleman SH, Abrams EJ, Bwakura-Dangarembizi M, et al Predictors of virologic and clinical response to nevirapine versus lopinavir/ritonavir-based antiretroviral therapy in young children with and without prior nevirapine exposure for the prevention of mother-to-child HIV transmission. Pediatr Infect Dis J. 2014;33(8):846–54. 25222305 10.1097/INF.0000000000000337 PMC4166566

[38] Sigaloff KC, Calis JC, Geelen SP, van Vugt M, de Wit TF HIV-1-resistance-associated mutations after failure of first-line antiretroviral treatment among children in resource-poor regions: a systematic review. Lancet Infect Dis. 2011;11(10):769–79. 21872531 10.1016/S1473-3099(11)70141-4

[39] Taylor BS, Hunt G, Abrams EJ, Coovadia A, Meyers T, Sherman G, et al Rapid development of antiretroviral drug resistance mutations in HIV-infected children less than two years of age initiating protease inhibitor-based therapy in South Africa. AIDS Res Hum Retroviruses. 2011;27:945–56. 21345162 10.1089/aid.2010.0205 PMC3161115

[40] Musiime V, Kaudha E, Kayiwa J, Mirembe G, Odera M, Kizito H, et al Antiretroviral drug resistance profiles and response to second-line therapy among HIV type 1-infected Ugandan children. AIDS Res Hum Retroviruses. 2013;29(3):449–55. 23308370 10.1089/aid.2012.0283

[41] Salou M, Dagnra AY, Butel C, Vidal N, Serrano L, Takassi E, et al High rates of virological failure and drug resistance in perinatally HIV-1-infected children and adolescents receiving lifelong antiretroviral therapy in routine clinics in Togo. J Int AIDS Soc. 2016;19(1):20683. 27125320 10.7448/IAS.19.1.20683 PMC4850147

[42] World Health Organisation Consolidated guidelines on the use of antiretroviral drugs for treating and preventing HIV infection. Recommendations for a public health approach. Geneva: WHO, UNAIDS; 2013 [cited 2013 630]. Available from: http://www.who.int/hiv/pub/guidelines/arv2013/download/en/index.html 24716260

[43] Cotton MF, Violari A, Otwombe K, Panchia R, Dobbels E, Rabie H, et al Early time-limited antiretroviral therapy versus deferred therapy in South African infants infected with HIV: results from the children with HIV early antiretroviral (CHER) randomised trial. Lancet. 2013;382(9904):1555–63. 24209829 10.1016/S0140-6736(13)61409-9 PMC4104982

[44] Castro H, Judd A, Gibb DM, Butler K, Lodwick RK, van Sighem A, et al Risk of triple-class virological failure in children with HIV: a retrospective cohort study. Lancet. 2011;377(9777):1580–87. 21511330 10.1016/S0140-6736(11)60208-0 PMC3099443

[45] Meyers T, Sawry S, Wong JY, Moultrie H, Pinillos F, Fairlie L, et al Virologic failure among children taking lopinavir/ritonavir-containing first-line antiretroviral therapy in South Africa. Pediatr Infect Dis J. 2015;34(2):175–79. 25741970 10.1097/INF.0000000000000544 PMC4352713

[46] UNAIDS, Joint United Nations, Programme on HIV/AIDS The 90-90-90: an ambitious treatment target to help end the AIDS epidemic 2014. Geneva: UNAIDS; 2014 [cited 2014 10]: [40 p.]. Available from: http://www.unaids.org/sites/default/files/media_asset/90-90-90_en_0.pdf updated September 2014 Report No.

